# MASS: predict the global qualities of individual protein models using random forests and novel statistical potentials

**DOI:** 10.1186/s12859-020-3383-3

**Published:** 2020-07-06

**Authors:** Tong Liu, Zheng Wang

**Affiliations:** Department of Computer Science, University of Miami, 1365 Memorial Drive, P.O. Box 248154, Coral Gables, FL 33124 USA

**Keywords:** Protein model quality assessment, Single-model QA, Random forests, Protein energy potentials

## Abstract

**Background:**

Protein model quality assessment (QA) is an essential procedure in protein structure prediction. QA methods can predict the qualities of protein models and identify good models from decoys. Clustering-based methods need a certain number of models as input. However, if a pool of models are not available, methods that only need a single model as input are indispensable.

**Results:**

We developed MASS, a QA method to predict the global qualities of individual protein models using random forests and various novel energy functions. We designed six novel energy functions or statistical potentials that can capture the structural characteristics of a protein model, which can also be used in other protein-related bioinformatics research. MASS potentials demonstrated higher importance than the energy functions of RWplus, GOAP, DFIRE and Rosetta when the scores they generated are used as machine learning features. MASS outperforms almost all of the four CASP11 top-performing single-model methods for global quality assessment in terms of all of the four evaluation criteria officially used by CASP, which measure the abilities to assign relative and absolute scores, identify the best model from decoys, and distinguish between good and bad models. MASS has also achieved comparable performances with the leading QA methods in CASP12 and CASP13.

**Conclusions:**

MASS and the source code for all MASS potentials are publicly available at http://dna.cs.miami.edu/MASS/.

## Background

The quality assessment (QA) of protein models plays an important role in protein tertiary structure prediction and model refinement [1]. Since it was introduced into the critical assessment of techniques for protein structure (CASP) as an independent category in 2006, various methods have been developed for predicting the qualities of protein models [2–8]. Computational quality assessment tools can be categorized into three types: single-model methods, clustering-based methods, and quasi-single methods. Compared with clustering-based methods that require a pool of protein models as input, single-model methods only need an individual protein model as input [8] and are indispensable when there are only a few models available in the model pool. Quasi-single methods can be thought of as a hybrid of single-model and clustering-based methods.

Most of the single-model methods are developed based on machine learning algorithms, such as support vector machine (SVM) [9, 10], random forests [11], and deep learning algorithms [12–15]. Single-model methods have used various features for training the machine learning models, such as energy functions [7, 11] and the consistency between predicted and assigned secondary structures [8]. Liu et al. [8] developed a deep learning architecture based on stacked denoising encoders (SdA) to predict residue-specific qualities of individual models. Cao et al. developed DeepQA [5], in which energy functions, physio-chemical characteristics, and structural information were used as features, and deep belief networks were used as the machine learning algorithm. ProQ3 [7] used Rosetta energy terms as input features and SVM as machine learning algorithm and outperformed its previous version ProQ2 [10].

In this study, we present a single-model method named MASS for predicting global qualities of individual protein models. We designed and re-implemented ten protein potentials and proved that they indicate different structural or energetic characteristics of a protein model. The random forests algorithm is used as the machine learning algorithm; the values from ten potentials along with six other types of features are used to predict the global qualities of individual models. We evaluated MASS along with other QA methods in CASP11, CASP12, and CASP13 and found that MASS outperforms most of the methods in CASP11 and is comparable with the leading methods in CASP12 and CASP13.

## Methods

### Training data and features

The training data were collected from previous CASP experiments: 85 targets from CASP9 and 67 from CASP10. The objective values are GDT-TS scores obtained from superimposing protein models with their native structures using LGA [16]. For each protein target, there are about 300 models. Considering the small differences between the five models from the same group, we randomly selected 150 models on each target for generating machine learning features.

We used 70 features in seven categories: (1) the agreement of predicted and assigned secondary structures, specifically, the Q3, SOV’99, SOV_refine scores [17] and three solvent accessibility scores (six features); (2) existing statistical potential energy of protein models, including RWplus [18], GOAP [19], and DRIRE [20] (three features); (3) pseudo amino acid composition of the amino acid sequences [21] (23 features); (4) radius of gyration of the models (one feature); (5) residue-residue contact information (two features); (6) newly-designed and newly-implemented protein statistical potentials (15 features); and (7) Rosetta energy functions [22] (20 features).

#### Comparison between predicted and assigned secondary structures and solvent accessibilities

The predicted secondary structures and relative solvent accessibilities were obtained by executing SCRATCH [23]. The secondary structures and relative solvent accessibilities of a protein model were assigned by STRIDE [24]. The Q3, SOV’99, and SOV_refine scores [17, 25] were used to assess the similarity between the predicted and assigned protein secondary structures. The other three features indicate the percentage of identical values between the predicted and assigned relative solvent accessibilities including buried, exposed, and both buried and exposed at 25% exposure threshold.

#### Statistical potential energy

We used three statistical potential energy scores, including the ones generated by RWplus [18], GOAP [19], and DFIRE [20].

#### Pseudo amino acid composition

Pseudo amino acid composition (PseAA) [21] was used to indicate amino acid composition.

#### Radius of gyration

Radius of gyration has been widely used as an indicator of protein structure compactness [26]. When we used radius of gyration in this study, we only considered N, Cα, and C atoms, and assumed that all atoms of interest have equal masses.

#### Residue-residue contact information

The in-contact relationship between the Cα-Cα atoms is defined as the sequence separation ≥ 6 and Euclidean distance in 3D space less than 8 Å. The first feature is the average sequence separation between atoms that are in-contact. The second feature is the average value of the distances between in-contact Cα-Cα atoms weighted by their sequence separations.

#### MASS potentials

We designed six protein statistical potentials from scratch including pseudo-bond angle potential (PAP), accessible surface potential at the atomic level (ASPA), sequence separation-dependent potential (SSDP), contact-dependent potential (CDP), relative solvent accessibility potential (RSAP), and volume-dependent potential (VDP). We re-designed (made minor modifications on the existing designs) the torsion angle potential (TAP) previously defined in QMEAN [27]. We re-implemented (the potentials were previously defined by others; we implemented them in PERL) three previously defined protein potentials: centrosymmetric burial potential (CSP), accessible surface potential at the residue level (ASPR), and distance-dependent potential (DDP).

We used both Cα and Cβ atoms to represent a residue in five potentials: ASPR, CDP, CSP, DDP, and SSDP. Therefore, we used in total 15 potentials for a given protein model (will be referred to as MASS potentials hereafter). The protein dataset we used for extracting reference states is TOP8000 [28], which contains about 8000 high-resolution (< 2.0 Å) and quality-filtered experimentally-determined protein structures (chains) with 70% PDB homology level. The dataset was previously used to update the torsional distributions in MolProbity [28] and was used here to extract the distributions of other reference states.

The reference state information consists of pseudo-bond angles, torsion angles, centrosymmetric burial, accessible surface at the residue level and at the atomic level, residue distance, sequence separation, residue-residue contact, relative solvent accessibility, and atom volume. The general formula [29, 30] we used to calculate the potentials of an atom (a residue) or paired atoms (paired residues) is:

$$ E= RT\times \ln \left[1+M\times \sigma \right]- RT\times \ln \left[1+M\times \sigma \times \frac{f_{observed}}{f_{reference}}\right] $$,

where σ is a weight parameter and was set to 1/50 [31] and *RT* was set to 0.582 kcal/mole [30]. For the potentials discussed below, we used newly-designed ways of calculating *M*, *f*_*observed*_, and *f*_*reference*_.

#### Pseudo-bond angle potential

We defined pseudo-bond angles as the angles formed by three consecutive N, Cα, or C atoms in the backbone. The 180° degree of pseudo-bond angles is evenly split into *n = 6* classes. The *M* value in the pseudo-bond angle potential (PAP) for a specific residue is defined as:

$$ M={M}_R=\sum \limits_{ss\in \left\{H,E,C\right\}}\sum \limits_{c_N=1}^6\sum \limits_{c_{C_{\alpha }}=1}^6\sum \limits_{c_C=1}^6f\left(R, ss,{c}_N,{c}_{C_{\alpha }},{c}_C\right) $$,

where *R* denotes the residue type, *ss* is the secondary structure state of the residue, *c*_*N*_ is the class of pseudo-bond angle formed by N atoms, ${c}_{C_{\alpha }}$ is the class of pseudo-bond angle formed by Cα atoms, *c*_*C*_ is the class of pseudo-bond angle formed by C atoms, and *f* is a function that returns the number of occurrences of a specific combination of *R*, *ss*, *c*_*N*_, *c*_*Cα*_, and* c*_*C*_. Therefore, for a specific residue type *R* its *M* is the number of observations for different secondary structure states and pseudo-bond angle classes based on our reference state information.

For the *i*th residue with residue type *R*_*i*_, suppose it has a specific combination of states $\left( ss,{c}_N,{c}_{C_{\alpha }},{c}_C\right)$, then we define ${f}_i\left( ss,{c}_N,{c}_{C_{\alpha }},{c}_C\right)$ as the number of occurrences of that combination of states for residue type *R*_*i*_ (one occurrence number is generated for a residue type, with in total 20 occurrence numbers generated for 20 amino acid types). We define *f*_*reference*_ as:

$$ {f}_{referene}=\frac{\sum_{R=1}^{20}{f}_i\left( ss,{c}_N,{c}_{C_{\alpha }},{c}_C\right)}{\sum_{R=1}^{20}{M}_R} $$,

where the denominator is the sum of all observations or occurrences for all residue types based on our reference state information, and the numerator is the sum of the observations for this specific state combination for all residue types. The *f*_*observed*_ is defined as:

$$ {f}_{observed}=\frac{f_i\left({R}_i, ss,{c}_N,{c}_{C_{\alpha }},{c}_C\right)}{M_{R_i}} $$

. *f*_*observed*_ is very similar to *f*_*reference*_, but when calculating the former one, we only considered the residue type *R*_*i*_.

#### Torsion angle potential

We refined the definition of torsion angle potential (TAP) previously defined in QMEAN [27]. For three adjacent residues in a protein chain, six dihedral angles (Φ_*i* − 1_, Ψ_*i* − 1_, Φ_*i*_, Ψ_*i*_, Φ_*i* + 1_, Ψ_*i* + 1_) are defined. For each of the six dihedral angles, we first evenly split the 360° degree into *n* = 9 classes. The existing definition of TAP includes two types of combinations of the six dihedral angels: (Φ_i-1_, Φ_i_, Φ_i+1_) and (Ψ_i-1_, Ψ_i_, Ψ_i+1_). We will use (a_1_, a_2_, a_3_) hereafter to indicate these two categories of combinations. For a given specific class set or combination (a_1_, a_2_, a_3_), we further created another two approaches of defining the state combinations. The first approach defines five classes *c*_5_: if a_1_ = a_2_ *and* a_1_ = a_3_, we label it as *c*_51_; if a_1_ ≠ a_2_, *a*_2_ ≠ *a*_3_, *and* a_1_ ≠ a_3_, we label it as *c*_52_; if a_1_ = a_2_ *and* a_1_ ≠ a_3_, we label it as *c*_53_; if a_1_ = a_3_ *and* a_1_ ≠ a_2_, we label it as *c*_54_; and if a_2_ = a_3_ *and* a_1_ ≠ a_2_, we label it as *c*_55_. The second apporach defines four classes *c*_4_: if a_2_ = a_1_ *and* a_2_ = a_3_, we label it as *c*_41_; if a_2_ ≠ a_1_ *and* a_2_ ≠ a_3_, we label it as *c*_42_; if a_2_ = a_1_ *and* a_2_ ≠ a_3_, we label it as *c*_43_; and if a_2_ = a_3_ *and* a_2_ ≠ a_1_, we label it as *c*_44_. Therefore, given a set of six dihedral angles (Φ_*i* − 1_, Ψ_*i* − 1_, Φ_*i*_, Ψ_*i*_, Φ_*i* + 1_, Ψ_*i* + 1_), we first classify each of them into *n* = 9 classes *c*(Φ_*i* − 1_, Ψ_*i* − 1_, Φ_*i*_, Ψ_*i*_, Φ_*i* + 1_, Ψ_*i* + 1_), and then classify them based on *c*_5_ and *c*_4_ if we set (a_1_, a_2_, a_3_) = *c*(Φ_*i* − 1_, Φ_*i*_, Φ_*i* + 1_) or (a_1_, a_2_, a_3_) = *c*(Ψ_*i* − 1_, Ψ_*i*_, Ψ_*i* + 1_). The refined definition of *M* for the torsion angle potential for a specific residue is:

$$ M={M}_R=\sum \limits_{ss\in \left\{H,E,C\right\}}\sum \limits_{{\Phi c}_5=1}^5\sum \limits_{\Psi {c}_5=1}^5\sum \limits_{{\Phi c}_4=1}^4\sum \limits_{\Psi {c}_4=1}^4f\left(R, ss,\Phi {c}_5,\Psi {c}_5,\Phi {c}_4,\Psi {c}_4\right) $$,

where Φ*c*_5_ denotes the category of Φ, that is, *c*(Φ_*i* − 1_, Φ_*i*_, Φ_*i* + 1_) and *c*_5_ class definition, similarly for Ψ*c*_5_, Φ*c*_4_, Ψ*c*_4_.

For the *i*th residue with residue type *R*_*i*_, suppose it has a specific state combination (*ss*, Φ*c*_5_, Ψ*c*_5_, Φ*c*_4_, Ψ*c*_4_ ), we then define *f*_*i*_(*ss*, Φ*c*_5_, Ψ*c*_5_, Φ*c*_4_, Ψ*c*_4_) as the number of occurrences of that combination of states for residue type *R*_*i*_ (one occurrence number is generated for a residue type, with in total 20 occurrence numbers generated for 20 amino acid types). We define *f*_*reference*_ and *f*_*observed*_ as: 

$$ {f}_{referene}=\frac{\sum_{R=1}^{20}{f}_i\left( ss,\Phi {c}_5,\Psi {c}_5,\Phi {c}_4,\Psi {c}_4\right)}{\sum_{R=1}^{20}{M}_R} $$

$$ {f}_{observed}=\frac{f_i\left({R}_i, ss,\Phi {c}_5,\Psi {c}_5,\Phi {c}_4,\Psi {c}_4\right)}{M_{R_i}} $$

The definitions of *M*_*R*_, *f*_*referene*_, and *f*_*observed*_ are very similar to the ones we defined in Pseudo-bond angle potential, but here we use a two-layer class assignment system (first use a_1_, a_2_, and a_3_ and then use c_4_ and c_5_) for torsion angles.

#### Centrosymmetric burial potential

We re-implemented the centrosymmetric burial potential (CSP) [32] with two major alternations. One of them is that we integrated the static radius of gyration (Rg) for protein models and native structures. The Rg we used in this work was derived from a simple function of the number of residues (N): Rg = 0.395 × N^0.6^ + 7.257, resulting from regression analysis between a dataset of about 1000 globular proteins from the PDB database and their corresponding sequence lengths [33]. The second alternation is that we added secondary structure classes (i.e., H, E, and C), in the same way as in the former two potentials. The range [0, 3 × *Rg*] is evenly divided into 30 bins or classes *c*_30_. For each atom (Cα or Cβ), we calculated the distance between the atom and center of mass of the current protein and determine which *c*_30_ this distance belongs to. The *M*,  f_reference_, and f_observed_ for the CSP for each atom (Cα or Cβ) are defined as:

$$ M={M}_R=\sum \limits_{ss\in \left\{H,E,C\right\}}\sum \limits_{c_{30}=1}^{30}f\left(R, ss,{c}_{30}\right) $$

$$ {f}_{referene}=\frac{\sum_{R=1}^{20}{f}_i\left( ss,{c}_{30}\right)}{\sum_{R=1}^{20}{M}_R} $$

$$ {f}_{observed}=\frac{f_i\left({R}_i, ss,{c}_{30}\right)}{M_{R_i}} $$

For the *i*th residue (atom Cα or Cβ) with residue type *R*_*i*_, suppose it has a specific state combination of (*ss*, *c*_30_), we define *f*_*i*_(*ss*, *c*_30_) as the number of occurrences of that combination of states for residue type* R*_*i*_ (one occurrence number is generated for a residue type, with in total 20 occurrence numbers generated for 20 amino acid types).

#### Accessible surface potential at the residue level

We re-implemented the accessible surface potential at the residue level (ASPR) [34]. The accessible surface of any given residue is calculated as the total number of residues locating within a 11 Å radius sphere centered on the given residue [34]. The accessible surface for a given residue is classified into 25 classes *c*_25_ (the range [0, 50] is evenly divided into 25 bins). The *M*, f_reference_, and f_observed_ for the ASPR for each residue (represented by Cα or Cβ atom) are defined as following:

$$ M={M}_R=\sum \limits_{ss\in \left\{H,E,C\right\}}\sum \limits_{c_{25}=1}^{25}f\left(R, ss,{c}_{25}\right) $$

$$ {f}_{referene}=\frac{\sum_{R=1}^{20}{f}_i\left( ss,{c}_{25}\right)}{\sum_{R=1}^{20}{M}_R} $$

$$ {f}_{observed}=\frac{f_i\left({R}_i, ss,{c}_{25}\right)}{M_{R_i}} $$

For the *i*th residue (represented by Cα or Cβ atom) with residue type *R*_*i*_, suppose it has a specific state combination (*ss*, *c*_25_), we define *f*_*i*_(*ss*, *c*_25_) as the number of occurrences of that combination of states for residue type *R*_*i*_ (one occurrence number is generated for a residue type, with in total 20 occurrence numbers generated for 20 amino acid types).

#### Accessible surface potential at the atomic level

We designed accessible surface potential at the atomic level (ASPA) based on the classification of all heavy atoms into 40 atom types *c*_40_ [35]. For each given heavy atom, we calculated its accessible surface as we did for ASPR but used an 8 Å radius sphere. The accessible surface for a given heavy atom is classified into 30 classes *c*_30_ (the range [50,200] is evenly divided into 30 bins). The *M*, f_reference_, and f_observed_ for the ASPA for each heavy atom are defined as:

$$ M={M}_R=\sum \limits_{c_{30}=1}^{30}f\left(R,{c}_{30}\right) $$

$$ {f}_{referene}=\frac{\sum_{R=1}^{40}{f}_i\left({c}_{30}\right)}{\sum_{R=1}^{40}{M}_R} $$

$$ {f}_{observed}=\frac{f_i\left({R}_i,{c}_{30}\right)}{M_{R_i}} $$

For the *i*th heavy atom with atom class *R*_*i*_, suppose it has a specific state *c*_30_ , we define  *f*_*i*_(*c*_30_) as the number of occurrences of that state for heavy atom type *R*_*i*_ (one occurrence number is generated for one type of heavy atoms, with in total 40 occurrence numbers generated for 40 heavy atom types).

#### Distance-dependent potential

We re-implemented the same distance-dependent potential (DDP) as described in [29, 30]. Therefore, no detailed description is shown here. We evenly divided the distance range [5, 25] into 40 classes and only considered any two residues with at least three residues away. When calculating DDP, we used Cα or Cβ atom to represent a residue.

#### Sequence separation-dependent potential

We designed sequence separation-dependent potential (SSDP) based on the definition of DDP. SSDP is very similar to DDP, but we evenly divided the sequence separation range [0,300] into 60 classes and only considered two residues with distances equal to or less than 8 Å. For calculating SSDP, we used Cα or Cβ atom to represent a residue.

#### Contact-dependent potential

We designed contact-dependent potential (CDP). Two residues, with sequence separation equal to or larger than 6, are considered to be in-contact if their Euclidean distance is less than 9 Å in the 3D space. Therefore, the *M*, f_reference_, and f_observed_ for the CDP for any two residues (each represented by their Cα or Cβ atom and each belonging to 20 residue-type classes *c*_*j*_) being in-contact are defined as:

$$ M={M}_R=\sum \limits_{ss\in \left\{H,E,C\right\}}\sum \limits_{c_j=1}^{20}f\left(R, ss,{c}_j\right) $$

$$ {f}_{referene}=\frac{\sum_{R=1}^{20}{f}_i\left( ss,{c}_j\right)}{\sum_{R=1}^{20}{M}_R} $$

$$ {f}_{observed}=\frac{f_i\left({R}_i, ss,{c}_j\right)}{M_{R_i}} $$

For the *i*th and *j*th residues (represented by Cα or Cβ atoms) with residue type *R*_*i*_ and *c*_*j*_, suppose they have a specific class combination (*ss*, *c*_*j*_), we define  *f*_*i*_(*ss*, *c*_*j*_) as the number of occurrences of that combination of states for residue type *R*_*i*_ (one occurrence number is generated for a residue type, with in total 20 occurrence numbers generated for 20 amino acid types).

#### Relative solvent accessibility potential

We designed relative solvent accessibility potential (RSAP) from scratch. The relative solvent accessibility is assigned by STRIDE [24], and evenly divided into 10 classes *c*_10_ from range [0, 1]. The *M*, f_reference_, and f_observed_ for the RSAP for each residue (represented by the Cα or Cβ atom) are defined as:

$$ M={M}_R=\sum \limits_{ss\in \left\{H,E,C\right\}}\sum \limits_{c_{10}=1}^{10}f\left(R, ss,{c}_{10}\right) $$

$$ {f}_{referene}=\frac{\sum_{R=1}^{20}{f}_i\left( ss,{c}_{10}\right)}{\sum_{R=1}^{20}{M}_R} $$

$$ {f}_{observed}=\frac{f_i\left({R}_i, ss,{c}_{10}\right)}{M_{R_i}} $$

For the *i*th residue (represented by the Cα or Cβ atom) with residue type *R*_*i*_, suppose it has a specific state combination (*ss*, *c*_10_), we define *f*_*i*_(*ss*, *c*_10_) as the number of occurrences of that combination of states for residue type *R*_*i*_ (one occurrence number is generated for a residue type, with in total 20 occurrence numbers generated for 20 amino acid types).

#### Volume-dependent potential

We designed a new potential: volume-dependent potential (VDP). For each Cα atom, we calculated its volume as described in [36]. Given a volume value, we classified it into 10 classes *c*_10_ (range [10, 30] was evenly divided). The *M*, f_reference_, and f_observed_ for the VDP for each residue (represented by Cα atom) are defined as:

$$ M={M}_R=\sum \limits_{ss\in \left\{H,E,C\right\}}\sum \limits_{c_{10}=1}^{10}f\left(R, ss,{c}_{10}\right) $$

$$ {f}_{referene}=\frac{\sum_{R=1}^{20}{f}_i\left( ss,{c}_{10}\right)}{\sum_{R=1}^{20}{M}_R} $$

$$ {f}_{observed}=\frac{f_i\left({R}_i, ss,{c}_{10}\right)}{M_{R_i}} $$

For the *i*th residue (represented by the Cα atom) with residue type *R*_*i*_, suppose it has a specific class combination (*ss*, *c*_10_), we define *f*_*i*_(*ss*, *c*_10_) as the number of occurrences of that combination of states for residue type *R*_*i*_ (one occurrence number is generated for a residue type, with in total 20 occurrence numbers generated for 20 amino acid types).

#### Rosetta energy functions

We used Rosetta Energy Function 2015 (REF15) to generate 19 energy scores for each residue [22]. The Rosetta energy of a protein model is the sum of all residues’ energy scores; and the twentieth energy score provided by REF15 is the sum of all 19 energy scores.

#### Optimal parameters

Given a protein model, we first calculated the ten potentials at the atom or residue level and obtained the potentials/energy scores of this protein model by summing up all residues’ energy scores (∑*E*). To determine the optimal parameters (e.g., range boundaries and class number) in each of the ten potentials, we selected 730 single-domain models in CASP9 by randomly selecting 10 models from each of the 73 template-based-modelling (TBM) single-domain targets. We tested various configurations of parameters. The Pearson’s and Spearman correlations between GDT-TS of the 730 models and their corresponding energy scores were used to determine the final parameters (see Additional file 1: Table S1).

### Random forests

The random forests algorithm [37] was used as the machine learning algorithm in this study, which has been widely used in the field of computational biology [38, 39]. We used five-fold cross-validation to determine the optimal parameters (i.e., ntree, mtry) [40]. The number of trees (i.e., ntree) we tested were from 500 to 5000 with an interval of 500. The mtry values we tested were from 10 to 34 with an interval of one. The optimal parameters we obtained were 2500 and 24, respectively.

## Results

Similar to how CASP officially evaluates QA methods that predict global qualities [1] of protein models, we assessed our method, together with four methods participated in CASP11, seven in CASP12, and 16 in CASP13, by four criteria measuring the abilities to assign relative scores, identify the best model from decoys, assign absolute scores, and discriminate good models from bad models. The corresponding measures are (1) the weighted mean of Pearson’s product moment correlation coefficient (wmPMCC), (2) the average loss (Ave loss), (3) the average GDT-TS deviations (Ave ΔGDT), and (4) the Matthews correlation coefficient (MCC) and receiver operating characteristic (ROC). The weighted mean of Pearson’s product moment correlation coefficient (wmPMCC) was used to evaluate the QA methods’ ability to predict relative model accuracy. In each stage, the Pearson’s correlation coefficients *r* between predicted and real GDT-TS scores for each target were calculated, and then the correlation *r* was transformed into an additive quantity using:

$$ \mathrm{z}=\frac{1}{2}\ln \left(\frac{1+r}{1-r}\right) $$,

where *z* is the normally distributed variable. We then calculated the arithmetic mean score of *z* values, denoted as $\overline{z}$. The final wmPMCC $\overline{r}$ was obtained using the following inverse equation.

$$ \overline{r}=\frac{e^{\overline{z}}-{e}^{-\overline{z}}}{e^{\overline{z}}+{e}^{-\overline{z}}} $$

The average loss (Ave loss) was designed to assess the quality of identifying the best model from a pool of models of each target. The loss value is the absolute value between the native GDT-TS scores of the best model and the predicted best model, which means that smaller loss values correspond to better ability to identify best models.

Compared with wmPMCC, the average GDT-TS deviation (Ave Δ GDT) was used to evaluate the QA methods’ ability to assign absolute model accuracy. For each model in a target, the GDT-TS deviation is the absolute value between real GDT-TS score and predicted global-quality score.

To evaluate the ability of distinguishing between good and bad models, we computed the Matthews correlation coefficient (MCC). For a protein model, if its true GDT-TS score is ≥50 (out of 100) and a QA method assigns a score ≥ 50, we counted it as true positive (TP). The MCC score was calculated as:

$$ \mathrm{MCC}=\frac{TP\times TN- FP\times FN}{\sqrt{\left( TP+ FP\right)\left( TP+ FN\right)\left( TN+ FP\right)\left( TN+ FN\right)}} $$,

where TN stands for true negatives, FP for false positives, and FN for false negatives. We also performed the receiver operating characteristic (ROC) analysis [41]. ROC curves (AUC) indicate the ability of binary classification of the model's quality; if real GDT-TS ≥50, the model quality is considered good, otherwise poor.

We first proved that the ten MASS potentials are significantly different from each other. We calculated the Pearson’s correlations of every two potentials for 730 models (see Additional file 1: Table S2 upper triangular) and the statistical significance of the differences at the 95% confidence level using paired t-tests (see Additional file 1: Table S2 lower triangular). We also calculated the Spearman correlations of every two potentials for 730 models (see Additional file 1: Table S3 upper triangular) and statistical significance of the differences at the 95% confidence level using paired Wilcoxon Signed-Rank tests (see Additional file 1: Table S3 lower triangular). From Additional file 1: Tables S2 and S3, we can conclude that the ten MASS potentials we designed and re-implemented are statistically and significantly different from each other.

**Fig. 1:**
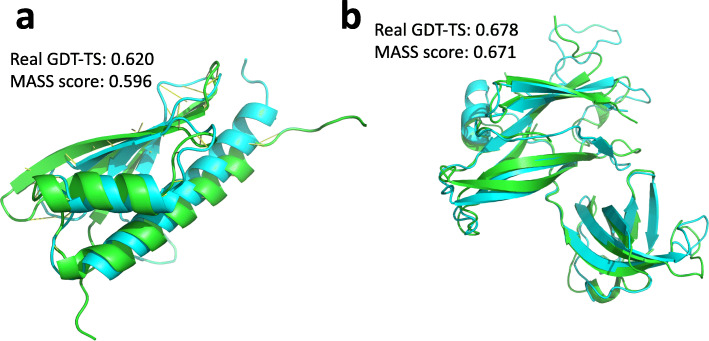
Two examples showing the real GDT-TS scores and MASS predicted scores. **a** The native 3D structure of T0882 in CASP12 (green) superimposed with the model of IntFOLD4_TS3 (blue). **b** The native structure of T0760 in CASP11 (green) superimposed with the model of Pcons-net_TS4 (blue)

We blindly tested MASS on 75 CASP11 targets in two stages (sel20 and best150), and compared its performance with four leading methods in CASP11: ProQ2 [3], ProQ2-refine [10], Qprob/ MULTICOM_NOVEL [4], and QAcon/MULTICOM-CLUSTER [42]. We also blindly tested MASS on 72 CASP12 targets and compared it with leading methods in CASP12 including ProQ3 [7], SVMQA [6], VoroMQA [43], DeepQA/MULTICOM-CLUSTER [5], Myprotein-me, QASproGP, and QMEAN [27]. As shown in Additional file 1: Table S4 for CASP11, our method (MASS) outperforms the other four in every aspect in stage 1, for stage 2, see Table 1. Our method is the only method that achieves > 0.7 wmPMCC in stage 1 and > 0.4 in stage 2, indicating that MASS can accurately predict the real GDT-TS scores. Figure 1 shows two example predictions of MASS on two CASP targets.

**Table 1:** Evaluations of our method MASS with four top-performing single-model methods in stage 2 for 75 targets of CASP 11 (groups are ranked by wmPMCC and best results are highlighted in bold)

Group ID	wmPMCC	Ave loss	Ave ΔGDT	MCC	ROC
MASS	**0.409**	0.07029	0.00076	0.60	**0.88**
QAcon	0.390	0.07543	**0.00064**	**0.61**	**0.88**
Qprob	0.368	0.07540	0.00113	0.52	0.86
ProQ2-refine	0.351	0.07068	0.00083	0.58	0.86
ProQ2	0.349	**0.06191**	0.00085	0.57	0.85

In terms of CASP12, as shown in Additional file 1: Table S5, ProQ3 and DeepQA outperform the others in stage 1, and ProQ3 and SVMQA outperform the others in stage 2, see Table 2. MASS outperforms SVMQA in terms of Ave ΔGDT in both stages and in terms of MCC and ROC in stage 1. Moreover, we reported the significance of differences between any two methods in Additional file 1: Table S6 for CASP 11 and Additional file 1: Table S7 for CASP 12 by Fisher Z-Transformation and t-test. It shows that the predictions of MASS are significantly different to the predictions of QAcon, ProQ3, and SVMQA.

**Table 2:** Evaluations of our method MASS with seven top-ranking single-model methods in stage 2 for 72 targets of CASP 12 (groups are ranked by wmPMCC and best results are highlighted in bold)

Group ID	wmPMCC	Ave loss	Ave ΔGDT	MCC	ROC
SVMQA	**0.677**	**0.05608**	0.00091	0.62	0.90
ProQ3	0.664	0.06073	0.00064	0.67	**0.93**
MASS	0.649	0.08744	0.00086	0.62	0.90
QASproGP	0.634	0.07992	0.00069	0.65	0.92
VoroMQA	0.619	0.08169	0.00077	0.16	0.86
DeepQA	0.616	0.08145	**0.00051**	**0.69**	**0.93**
Myprotein-me	0.614	0.10350	0.00089	0.44	0.80
QMEAN	0.311	0.10546	0.00141	0.41	0.81

We also evaluated our method using 57 targets in CASP13 experiment along with 16 methods participating in CASP13 including ModFOLD7 series [15], FaeNNz, ProQ4 [14], MESHI series, VoroMQA series [44], MULTICOM-NOVEL, Bhattacharya-SingQ, Bhattacharya-Server, PLU-AngularQA [45], and PLU-TopQA (methods having missing models or targets were excluded). The results are shown in Table 3 for stage 2 and Additional file 1: Table S8 for stage 1. In stage 1, ModFOLD7 series [15] perform better than the others according to the five evaluation metrics. MASS achieves a slightly lower ROC (i.e., 0.94) compared with 0.99 from ModFOLD7. In stage 2, ModFOLD7 series still outperform the other methods.

**Table 3:** Evaluations of our method MASS with seven top-ranking single-model methods in stage 2 for 57 targets of CASP 13 (groups are ranked by wmPMCC and best results are highlighted in bold)

Group ID	wmPMCC	Ave loss	Ave ΔGDT	MCC	ROC
ModFOLD7	**0.906**	0.0936	0.00041	0.72	**0.94**
ModFOLD7_cor	0.888	0.09313	**0.00039**	**0.74**	**0.94**
ModFOLD7_rank	0.839	**0.05807**	0.00091	0.64	0.93
FaeNNz	0.78	0.09127	0.00083	0.58	0.89
ProQ4	0.773	0.08708	0.00106	0.57	0.86
MESHI-enrich-server	0.756	0.08826	0.00087	0.52	0.88
MESHI-corr-server	0.742	0.08727	0.00088	0.57	0.88
VoroMQA-A	0.721	0.08322	0.00098	0.34	0.87
MUFold_server	0.714	0.08675	0.00095	0.6	0.89
VoroMQA-B	0.69	0.07854	0.001	0.33	0.86
MASS	0.682	0.09037	0.00106	0.54	0.85
MULTICOM-NOVEL	0.667	0.07839	0.00113	0.38	0.83
MASS2	0.652	0.09748	0.00124	0.46	0.83
Bhattacharya-SingQ	0.638	0.08676	0.00097	0.46	0.81
Bhattacharya-Server	0.601	0.11021	0.00106	0.44	0.82
PLU-AngularQA	0.57	0.13504	0.00097	0.44	0.83
PLU-TopQA	0.026	0.20285	0.00165	0.21	0.65

Notice that the pseudo amino acid composition for all models of a target are the same. In section 1 of the Additional file 1, we provided a discussion showing that although this feature cannot distinguish the models within a target, it can affect the scores given to all the models of a target.

**Fig. 2:**
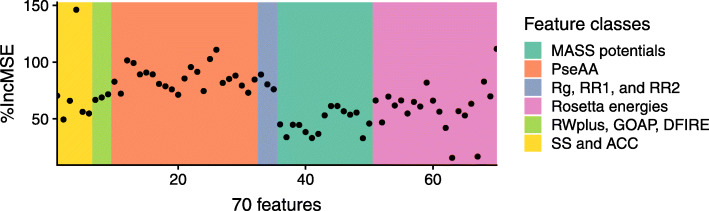
The feature importance of 70 features used in MASS. Different feature classes are highlighted with different colors

We provided the contribution of each of the 70 machine learning features in Fig. 2, which provides useful information for future research in this field. All of the 70 features play a positive role in the machine learning task with one of the solvent accessibility features, some of the PseAA features, and the twentieth energy scores from Rosetta contributing more than the rest.

The running time analysis of MASS is shown in the Additional file 1. Finally, to assess the values of the three energy sets including the three energy functions (RWplus, GOAP, and DFIRE), our novel MASS potentials, Rosetta energy functions, we individually occluded each of the three energy sets by setting the corresponding features to zero and then executed the same MASS model to obtain new predictions on 75 targets in CASP11 stage 2. We compared the evaluation results with/without occlusion and the results were shown in Additional file 1: Table S9. MASS potentials demonstrated higher importance than the three energy functions (RWplus, GOAP, and DFIRE) and Rosetta energy functions.

## Discussions

MASS is a random-forests-based approach for estimating the quality of individual protein models. It uses various features extracted from protein sequences and models. The features can be classified into seven sets: (1) consistency between predicted and assigned secondary structures and solvent accessibilities; (2) three energy functions (RWplus, GOAP, and DRIRE); (3) PseAA coding of protein sequence; (4) radius of gyration of the protein model; (5) residue-residue contact information; (6) 15 MASS potentials; and (7) 20 Rosetta energy functions. We evaluated MASS along with other QA methods in CASP11, CASP12, and CASP13. MASS outperforms most of the methods in CASP11 and is comparable with the leading methods in CASP12 and CASP13.

We defined and re-implemented 10 protein potentials using various protein properties including sequence-separation-dependent, distance-dependent, contact-dependent, volume-dependent, torsion angle, pseudo-bond angle, accessible surface, relative solvent accessibility, and centrosymmetric burial. We have proved that these 10 protein potentials play a key role in the good performance of MASS. The 10 MASS potentials can be used as machine learning features for other studies in the field of protein science, such as protein structure prediction and protein function prediction.

Currently, MASS does not support residue-specific (local) quality assessment, which can be used in refining protein models. However, most of the features we used in this work are residue-specific, which can be directly used as local or residue-specific features for developing residue-specific quality assessment methods. As our future work, we plan to integrate MASS potentials with deep learning methods to estimate residue-specific protein model qualities.

## Conclusions

In this study, we designed and implemented ten potentials using different reference state information including pseudo-bond angles, torsion angles, centrosymmetric burial, accessible surface at the residue level and at the atomic level, residue distance, sequence separation, residue-residue contact, relative solvent accessibility, and atom volume. We proved that the ten potentials were statistically significant different to each other. MASS potentials demonstrated higher importance than the three energy functions (RWplus, GOAP, and DFIRE) and Rosetta energy functions when used as machine learning features.

We also present MASS, which uses seven types of features and random forests to predict global qualities of individual protein models. To evaluate MASS and the related tools, we used four CASP-official-evaluation criteria that measured the abilities to assign relative and absolute scores, identify the best model from decoys, and distinguish between good and bad models. MASS outperforms almost all of the four CASP11 leading single-model methods for global quality assessment. MASS is comparable with most of the leading methods in CASP12 and CASP13 experiments.

## Supplementary information

**Additional file 1.** Supplementary Information and data. This document provides more details regarding pseudo amino acid composition, MASS potential comparision, and evaluation results for different QA methods in stage 1.

## Data Availability

MASS is publicly available at http://dna.cs.miami.edu/MASS/.

## Abbreviations

CASP: Critical assessment of techniques for protein structure prediction
QA: Quality assessment
