# Supplementary material for: MASS: predict the global qualities of individual protein models using random forests and novel statistical potentials
Source: BMC Bioinformatics. 2020 Jul 6;21(Suppl 4):246. doi: 10.1186/s12859-020-3383-3 (PMC7336608; doi:10.1186/s12859-020-3383-3)
Supplement: Supplementary file 1 — Additional file 1. Supplementary Information and data. This document provides more details regarding pseudo amino acid composition, MASS potential comparision, and evaluation results for different QA methods in stage 1. [file 12859_2020_3383_MOESM1_ESM.pdf]

MASS: Predict the global qualities of individual protein models using random forests and novel statistical potentials

Supplementary materials

1 Pseudo amino acid composition

We noticed that for all protein models of a given target this feature is the same. Therefore, we did a test to explore whether this feature is useful. For each of the 75 targets in CASP11 experiment we used in this study for blindly testing, we calculate its prediction difficulty level by averaging GDT-TS of 20 protein models in stage 1. We then select 10 targets with largest average GDT-TS and another 10 targets with smallest average GDT-TS. The distribution of the average GDT-TS for the two target sets can be found in Figure S1. For each of the two target sets, we calculate the Pearson correlation coefficients between any two different target sequences' PseAA. The distribution of the Pearson correlation coefficients shown in Figure S1 indicates that targets that are relatively easy to predict have more similar PseAA between each other than targets that are hard to predict. Therefore, although this feature cannot distinguish the models within a target, it can affect the scores given to all models of a target. For example, because of a certain amino acid sequence a target has, all the models of that target may be predicted with a relatively higher score (or lower if it is a hard target).

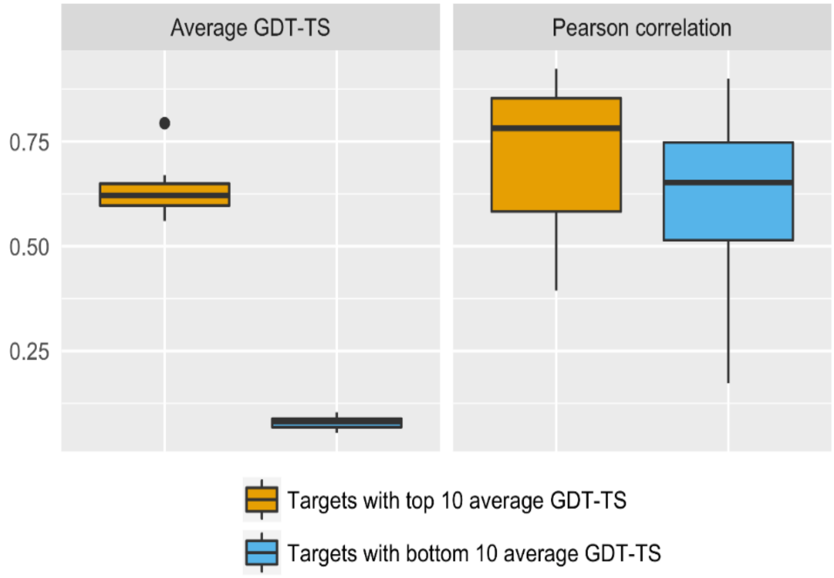

Figure S1. The average GDT-TS distribution of 10 targets with largest average GDT-TS and another 10 targets with smallest average GDT-TS. The Pearson correlation distribution between any two targets' PseAA in each of two target sets.

2 Running time

From our experience the computational resource and running time of single-model QA methods mainly depend on how they extract features from individual protein models. For example, our method MASS and ProQ series have a feature about comparison between predicted and assigned secondary structure; predicting secondary structures using SCRATCH (taking about three minutes for a protein model with 70 residues) is the main time-consuming process in running MASS. Therefore, given an individual protein model (e.g., 70 residues) MASS can predict its quality in about three minutes.

Table S1. The Pearson and Spearman correlations between GDT-TS and potential scores of the 730 protein models when we use the optimal parameters.

ID	Potential	Number of models	Pearson correlation	Spearman correlation
1	PAP	729	0.474	0.470
2	TAP (QMEAN)	729	0.332	0.351
	TAP	729	0.467	0.467
3	CSP-Cβ	701	0.536	0.471
	CSP-Cα	729	0.511	0.462
4	ASPR-Cβ	701	0.607	0.563
	ASPR-Cα	729	0.591	0.554
5	ASPA	730	0.605	0.600
6	DDP-Cβ	701	0.583	0.581
	DDP-Cα	730	0.522	0.503
7	SSDP-Cβ	701	0.495	0.529
	SSDP-Cα	730	0.428	0.466
8	CDP-Cβ	701	0.528	0.556
	CDP-Cα	729	0.520	0.514
9	RSAP	729	0.610	0.588
10	VDP	729	0.550	0.538

Table S2. Pearson correlations of any two potentials (upper triangular, r ≥ 0.8 highlighted) and statistical significance of the differences at the 95% confidence level between pairwise potentials using paired t-tests (lower triangular, p-value ≥ 0.05 highlighted).

	PAP	TAP	CSP	ASPR	ASPA	DDP	SSDP	CDP	RSAP	VDP
PAP	-	**0.85**	0.65	0.70	0.46	0.64	0.45	0.59	0.70	0.47
TAP	<0.05	-	0.71	0.69	0.44	0.57	0.43	0.58	0.70	0.44
CSP	<0.05	**0.93**	-	**0.82**	0.57	0.53	0.43	0.54	0.78	0.58
ASPR	<0.05	<0.05	<0.05	-	0.77	0.70	0.54	0.65	**0.93**	0.62
ASPA	<0.05	<0.05	<0.05	<0.05	-	0.61	0.48	0.53	**0.80**	0.66
DDP	<0.05	<0.05	<0.05	<0.05	<0.05	-	0.58	0.67	0.68	0.48
SSDP	<0.05	<0.05	<0.05	<0.05	<0.05	<0.05	-	0.34	0.54	0.40
CDP	<0.05	<0.05	<0.05	**0.63**	<0.05	<0.05	<0.05	-	0.61	0.46
RSAP	<0.05	<0.05	<0.05	<0.05	<0.05	<0.05	<0.05	**0.36**	-	0.61
VDP	<0.05	<0.05	<0.05	<0.05	<0.05	<0.05	<0.05	<0.05	<0.05	-

Table S3. Spearman correlations of any two potentials (upper triangular, r ≥ 0.8 highlighted) and statistical significance of the differences at the 95% confidence level between pairwise potentials using paired Wilcoxon Signed-Rank tests (lower triangular, p-value ≥ 0.05 highlighted).

	PAP	TAP	CSP	ASPR	ASPA	DDP	SSDP	CDP	RSAP	VDP
PAP	-	**0.81**	0.67	0.68	0.49	0.59	0.45	0.54	0.67	0.59
TAP	<0.05	-	0.69	0.67	0.48	0.54	0.44	0.54	0.67	0.57
CSP	<0.05	**0.29**	-	**0.81**	0.58	0.58	0.46	0.58	0.78	0.61
ASPR	<0.05	<0.05	<0.05	-	**0.80**	0.70	0.55	0.64	**0.92**	0.69
ASPA	<0.05	<0.05	<0.05	<0.05	-	0.60	0.48	0.54	**0.83**	0.64
DDP	<0.05	<0.05	<0.05	<0.05	<0.05	-	0.59	0.68	0.67	0.57
SSDP	<0.05	<0.05	<0.05	<0.05	<0.05	<0.05	-	0.36	0.57	0.46
CDP	<0.05	<0.05	<0.05	**0.93**	<0.05	<0.05	<0.05	-	0.60	0.52
RSAP	<0.05	<0.05	<0.05	<0.05	<0.05	<0.05	<0.05	**0.10**	-	0.68
VDP	<0.05	<0.05	<0.05	<0.05	<0.05	<0.05	<0.05	<0.05	<0.05	-

Table S4. Evaluations of our method MASS with four top-performing single-model methods in stage 1 for 75 targets of CASP 11 (Groups ranked by wmPMCC and best results highlighted).

Group ID	wmPMCC	Ave loss	Ave ΔGDT	MCC	ROC
MASS	**0.712**	**0.08436**	**0.00630**	**0.62**	**0.93**
QAcon	0.689	0.10073	0.00736	0.61	0.92
ProQ2-refine	0.689	0.09502	0.00688	0.56	0.91
ProQ2	0.672	0.09128	0.00690	0.54	0.90
Qprob	0.664	0.10203	0.01030	0.55	0.90

Table S5. Evaluations of our method MASS with seven top-ranking single-model methods in stage 1 for 72 targets of CASP 12 (Groups ranked by wmPMCC and best results highlighted).

Group ID	wmPMCC	Ave loss	Ave ΔGDT	MCC	ROC
DeepQA	**0.745**	0.07289	0.00515	0.49	0.96
Myprotein-me	0.719	0.09297	0.00619	0.21	0.89
ProQ3	0.700	0.04467	**0.00475**	**0.60**	**0.97**
SVMQA	0.679	**0.03644**	0.00809	0.36	0.92
MASS	0.653	0.07600	0.00763	0.45	0.95
VoroMQA	0.647	0.08048	0.00524	0.10	0.91
QASproGP	0.636	0.09425	0.00587	0.50	0.96
QMEAN	0.360	0.21315	0.01355	0.22	0.86

Table S6. Pairwise significance of difference on Fisher Z transformation from Pearson correlations between two predictions using t-test in stage 2 on CASP11 data set (P values provided).

	MASS	ProQ2	ProQ2-refine	Qprob	QAcon
MASS	-	2.79e-35	9.09e-35	1.43e-31	7.64e-33
ProQ2	-	-	9.05e-53	5.59e-43	1.58e-41
ProQ2-refine	-	-	-	1.1e-40	1.44e-40
Qprob	-	-		-	7.78e-46
QAcon	-	-	-	-	-

Table S7. Pairwise significance of difference on Fisher Z transformation from Pearson correlations between two predictions using t-test in stage 2 on CASP12 data set (P values provided).

	MASS	ProQ3	SVMQA	DeepQA	VoroMQA	Myprotein-me	QASproGP	QMEAN
MASS	-	6.18e-37	1.58e-35	1.28e-38	2.53e-35	2.31e-15	1.71e-36	7.35e-26
ProQ3	-	-	3.66e-41	1.82e-43	3.79e-46	5.85e-19	2.57e-38	1.22e-38
SVMQA	-	-	-	5.56e-41	2.1e-45	7.33e-19	7.1e-33	1.84e-39
DeepQA	-	-	-	-	1.53e-46	9.43e-18	5.11e-38	6.44e-37
VoroMQA	-	-	-	-	-	7.06e-21	5.66e-36	9.97e-42
Myprotein-me	-	-	-	-	-	-	6.2e-16	2.12e-12
QASproGP	-	-	-	-	-	-	-	1.05e-27
QMEAN	-	-	-	-	-	-	-	-

Table S8. Evaluations of our method MASS with seven top-ranking single-model methods in stage 1 for 57 targets of CASP 13 (Groups ranked by wmPMCC and best results highlighted).

GroupID	wmPMCC	Ave loss	Ave ΔGDT	MCC	ROC
ModFOLD7	**0.946**	0.00365	**0.00278**	**0.76**	**0.99**
ModFOLD7_cor	0.94	0.00514	0.00307	**0.76**	**0.99**
FaeNNz	0.832	0.02125	0.00641	0.47	0.96
ModFOLD7_rank	0.83	**0.00074**	0.00667	0.59	0.98
Bhattacharya-Server	0.795	0.08374	0.00462	0.41	0.82
Bhattacharya-SingQ	0.783	0.08156	0.00479	0.43	0.8
MULTICOM-NOVEL	0.769	0.04926	0.00486	0.36	0.81
ProQ4	0.768	0.0156	0.0075	0.45	0.85
MESHI-corr-server	0.757	0.05845	0.0067	0.52	0.95
MESHI-enrich-server	0.725	0.03438	0.00729	0.45	0.95
MASS	0.721	0.0582	0.00605	0.49	0.94
MUFold_server	0.71	0.03975	0.00786	0.48	0.94
VoroMQA-A	0.642	0.05738	0.00648	0.26	0.89
VoroMQA-B	0.638	0.0573	0.00661	0.35	0.87
MASS2	0.603	0.10557	0.01092	0.41	0.86
PLU-AngularQA	0.54	0.20311	0.0071	0.38	0.92
PLU-TopQA	0.159	0.32781	0.01825	0.14	0.73

Table S9. Evaluation of the value of the three energy sets, including the three energies (RWplus, GOAP, and DFIRE), our proposed MASS potentials, Rosetta energies on 75 targets in CASP 11 stage 2 by occluding the three energy sets individually (biggest changes highlighted).

Group ID	wmPMCC	Ave loss	Ave ΔGDT	MCC	ROC
MASS	0.409	0.07029	0.00076	0.60	0.88
Occluding RWplus, GOAP, and DFIRE	0.408	0.07882	0.00078	0.60	0.87
Occluding MASS potentials	**0.383**	**0.08056**	**0.00092**	**0.55**	**0.86**
Occluding Rosetta energies	0.406	0.07507	0.00084	**0.55**	**0.86**
